# Urine-Derived Stem Cells: The Present and the Future

**DOI:** 10.1155/2017/4378947

**Published:** 2017-11-08

**Authors:** Xiaoli Ji, Min Wang, Fang Chen, Junmei Zhou

**Affiliations:** ^1^Department of Central Laboratory, Shanghai Children's Hospital, Shanghai Jiao Tong University, 1400 Beijing Road West, Shanghai 200040, China; ^2^Department of Urology, Shanghai Children's Hospital, Shanghai Jiao Tong University, 1400 Beijing Road West, Shanghai 200040, China

## Abstract

Stem cell research provides promising strategies in improving healthcare for human beings. As a noninvasively obtained and easy-to-culture cell resource with relatively low expense, urine-derived stem cells have special advantages. They have been extensively studied on its proliferation ability and differentiation potential and were being reprogrammed to model diseases during the last decade. In this review, we intend to summarize the latest progress on the research of urine-derived stem cells for its broad application mainly in regenerative medicine and disease modeling, as well as in what is challenging currently. This minireview will highlight the potential application of urine-derived stem cells and provides possible direction of further research in the future.

## 1. Introduction

Stem cell research has remained to be an exploding and exciting area possessing the potential of improving healthcare for human beings [1, 2]. Tremendous research has been conducted on two types of stem cells: the pluripotent stem cells (PSCs) and the somatic stem cells. The most commonly investigated PSCs include embryonic stem cells (ESCs) and induced pluripotent stem cells (iPSCs). ESCs are usually derived from the inner cell mass of early embryos which could be proliferated for a long term and differentiated into cell types of all three germ layers *in vitro* and *in vivo* [3]. Based on the research of ESCs, mammalian somatic cells were reprogrammed to iPSCs by enforced expression of OCT3/4, SOX2, KLF4, and c-MYC [4] or an alternative set of OCT3/4, SOX2, LIN28, and NANOG [5]. The iPSCs, which bypass the ethical issue of ESCs resulting from destroying early embryos, could generate patient-specific cell types of various lineages *in vitro*. However, some obstacles including long-term manipulation, low reprogramming and differentiation efficiency, and tumorigenicity have prevented iPSCs from a broad range of clinical application. Meanwhile, the somatic stem cells could also be propagated to a large number of differentiated cell types, without the risk of tumor formation, thus enabling them to be closer to the clinical application compared with PSCs.

For the somatic stem cells, bone marrow-derived stem cells (BMSCs) and adipose-derived stem cells (ADSCs) are investigated for a long period and have been applied in various experimental studies and preclinical trials [6–9]. Although both BMSCs and ADSCs have multiple potential to differentiate into various cell types, they are obtained through invasive procedures and could bring damage to the patients, especially to the pediatric patients and those with abnormal hemorrhagic diseases. Therefore, within the latest decade, urine-derived stem cells (UDSCs) are emerging as a promising cell resource for their noninvasive obtaining procedure, potent proliferation ability, multiple application in cell therapy, and tissue engineering [10–15] and serve as original cells for reprogramming into disease-specific iPSCs [16]. Based on these applications, UDSCs are currently playing an important role in adult stem cell biology.

## 2. Multiple Transdifferentiation of UDSCs Applied in Cell Therapy and Tissue Engineering

The autologous somatic stem cells have special advantages for the future clinical application, since they usually do not induce immune rejection. In addition, the ability to expand to a large amount and to be induced into various cell lineages is also the basis for the somatic stem cells to explore their application in cell-based therapies and regenerative medicine. UDSCs could be expanded to yield a large population, and their plasticity has also been fully confirmed through investigation [17].

First, urine-derived cells were cultured from newborn children and displayed limited proliferation potential [18]. Urine cells with high proliferation ability were successfully cultured in a study of urological tissue reconstruction in 2008 [10]. In total, 55 urine samples from 15 volunteers and 8 patients were collected and cultured. Three types of cells with different morphologies were propagated representing fully differentiated, differentiating, and progenitor-like cells, respectively. Only the small cell type with a spindle appearance was named as UDSCs, as they could be consecutively proliferated for up to 20 passages, reaching accumulated population doubling (PD) rate of more than 60 [10, 15]. These cells most likely came from the parietal cell-podocyte interface of the renal glomerulus and expressed the corresponding markers [11, 12, 15]. Once isolated, the UDSCs could be consecutively expanded in culture *in vitro* and give rise to a variety of cell types via induction of lineage-specific differentiation under appropriate experimental conditions. Since its identification, up to now, UDSCs have been induced into ectodermal, mesodermal, and endodermal lineages. Ectodermal neural lineage was obtained through culturing UDSCs in neural induction medium supplemented with basic fibroblast growth factor [15, 19, 20]. Approximately 40% of the induced cells expressed several neural markers such as nestin, S100, NF200, and GFAP, as well as exhibiting neurogenic extensions and processes, both *in vitro* and *in vivo* [15, 19]. Human urine cells from volunteers and Wilson's disease patient could also be induced into neural lineage through the overexpression of Ascl1, Brn2, NeuroD, c-Myc, and Myt1l, characterized by expressing multiple neuronal markers and generating action potentials [20]. The neural lineage differentiation of UDSCs needs to be further investigated in future research. Endodermal lineage was obtained through culturing UDSCs in endothelial basal medium supplemented with vascular endothelial growth factor (VEGF). The induced cells developed a cobblestone-like morphology and expressed urothelial-specific markers such as uroplakin-III, uroplakin-Ia, CK7, and AE1/AE3 [15]. UDSCs have also been induced into multiple mesodermal lineage including osteogenic cells [21–23] and muscle cells [24–26]. After seeding on composite PLGA/CS scaffolds which were incorporated with calcium silicate, UDSCs demonstrated therapeutic potential in bone tissue regeneration *in vivo* through activation of the Wnt/*β*-catenin signaling pathway [22]. The UDSCs overexpressing VEGF could enhance the survival of grafted cells and promote myogenic differentiation, as well as improving the innervations, which could help to develop cell therapeutic strategy to correct stress urinary incontinence [26].

Tissue engineering is a promising field offering the possibility of providing scaffolds in order to structurally and functionally restore the altered pathological tissues. Although the complex structure and functions of the bladder have made this process challenging, great advancements have been achieved in the last few years using a scaffold and various stem cells to construct bladder and other urological tissues. Obtaining large amount of patients' cells is the primary issue in constructing autologous tissues. The ideal cells should be propagated through noninvasive manipulation and possess high proliferation ability. As a noninvasive and easy-to-expand cell resource, UDSCs have been applied in urological tissue engineering including bladder tissue engineering and urethral reconstruction [10, 15, 27–34]. The recent and previous publications have demonstrated that the autologous UDSCs could be differentiated into urothelial cells and smooth muscle cells. Besides, it could also be applied in urethral reconstruction with the advantages of less inflammation and fibrosis compared with the control group in urethral defect models [10, 27–29]. UDSCs have also been applied in bladder tissue engineering after being transdifferentiated into bladder-associated cell types such as smooth muscle cells and urothelial cells. Even more, they demonstrated markers such as tight junction including ZO-1, E-cadherin, and cingulin, which indicated that a protective ultrastructure barrier has formed and could probably protect the engineered bladder tissues from urine [15, 32]. Although there are still tremendous issues such as vascularization and neurotization which need to be settled before clinical application, these efforts have provided great potential for the use of UDSCs. In addition, more applications of UDSCs on nonurological tissue engineering need to be explored and investigated in the future.

## 3. UDSCs Served as Original Cells for Reprogramming into Disease-Specific iPSCs

Although animal models are commonly applied to investigate disease mechanisms, these *in vivo* research models have a few limitations which could be potentially overcome through ex vivo human cellular models such as iPSCs. Modeling various human diseases “in a culture dish” is a fundamental application of human disease-specific iPSCs for its genetic background of the targeted disease [16, 35–37].

Two steps including derivation of iPSCs from a patient's somatic cells and subsequent differentiation into disease-related cell types are important in modeling human diseases. Typically, parental somatic cells such as fibroblast and blood cells are harvested invasively from patients through biopsy or blood extraction. For some special patients such as children or those with abnormal hemorrhagic diseases, UDSCs have special advantages as they could be obtained noninvasively and cultured easily. Thus, UDSCs have been selected as alternative starting cells to generate iPSCs for both genders and all ages [38–40].

UDSC-derived disease-specific iPSCs have already been established in cardiac diseases [16], endocrine diseases [41, 42], abnormal hemorrhagic diseases resulting from various causes [43–45], aneuploidy diseases such as Down syndrome [46], neural diseases [47, 48], muscular disorders [49, 50], fibrodysplasia ossificans progressiva [51, 52], systemic lupus erythematosus [53], cryptorchidism [54], hypercholesterolemia [55], paroxysmal kinesigenic dyskinesia [56], and so on (Table 1). After successful reprogramming and characterization, differentiation experiments are essential, since most of disease phenotypes are usually observed in lineage-committed cells after *in vitro* differentiation rather than being observed in the iPSCs. *In vitro*, stepwise-directed differentiation is usually conducted according to the *in vivo* developmental pathway of the targeted cell type and often spans multiple weeks. Marker expression is detected during the consecutive developmental stage of differentiation both at mRNA level and at protein level. Even more, functional assays such as electrophysiology are also needed to study the pathophysiology of the targeted cells.

Based on these abovementioned research strategies, UDSC-derived iPSCs and the subsequent functional experiments have been applied in several disease modeling techniques. For the hemorrhagic disease category, iPSCs were generated successfully from 7 hemophilia A patients. The differentiated hepatocytes from these iPSCs failed to produce FVIII, which recapitulated the FVIII deficiency of hemophilia A. Thus, this cell model provided an effective way for modeling hemophilia A *in vitro* for further gene and cell therapy studies [45].

For the neurological disease category, urine samples were collected from 10 individuals with Down syndrome comprising 5 females and 5 males. The iPSCs were established and named as T21-iPSCs which were more sensitive to proteotoxic stress than euploid iPSCs. This study also indicated that T21-iPSCs could be differentiated into glutamatergic neurons which could fire action potential similar to euploid iPSCs. T21-iPSCs could also be induced into cardiomyocytes which exhibited spontaneous contractions and were sensitive to the beta adrenergic agonist isoproterenol [46]. Since both neurological disorders and congenital heart defects were the two most common complications of Down syndrome, these researches could probably be applied in human cell-based high-throughput drug screening in translational preclinical studies aimed at improving the life quality of patients with Down syndrome. Meanwhile, UDSCs have also been applied in the research of rare diseases. The long QT syndrome is a genetically inherited cardiac disease that can cause potentially fatal cardiac arrhythmia. Research showed that hiPSC derived from the HERG A561P-mutated urine cells (A561P-UhiPS CMs) can be differentiated into functional cardiomyocytes. Compared with the control healthy UhiPS-CMs, the A561P mutation caused a trafficking defect which led to delayed rectifier K^+^ current [16]. Fibrodysplasia ossificans progressiva (FOP) is an extremely rare connective tissue disease without effective treatment currently. It is characterized by progressive heterotopic ossification of soft tissues, and the molecular mechanisms underlying the pathology of FOP need to be investigated, as well as the identification of new therapeutic drugs through a proper research model [51]. The FOP-iPSC lines containing ALK2 mutation displayed decreased differentiation efficiency into bone-forming progenitors and reduced expression of VEGF receptor 2 in differentiated endothelial cells. The ALK2 kinase inhibitor could also partly inhibit the increase in mineralization of FOP-hiPSC-derived pericytes [52]. All these achievements had enabled the FOP-iPSCs as an alternative research model to evaluate the bioactivity of ALK2 inhibitors and other therapeutic drug candidates. Cryptorchidism is a common congenital birth defects, and infertility is an important complication with no proper treatment in adulthood [57]. Cryptorchidism-specific iPSCs have been established and differentiated into VASA-positive germ cell lineage which provided a potential model for investigating the mechanisms and treatments to infertility [54]. The autosomal dominant hypercholesterolemia is caused by mutated proprotein convertase subtilisin/kexin type 9 (PCSK9), which is a critical modulator of cholesterol homeostasis. PCSK9-iPSC lines were successfully established from urine cells and could be differentiated into hepatocyte-like cells. This study also indicated that the induced hepatocyte-like cells displayed altered PCSK9 secretion and LDL uptake, which mimic the pathophysiology of hypercholesterolemia and could be applied in drug screening [55]. Except for these abovementioned disease modeling techniques with functional experiments, further functional experiments need to be conducted on other UDSC-derived iPSCs after being induced into target cell types [41–44, 48–50, 53].

## 4. Current Challenges and Future Perspectives

UDSCs have been applied as a novel noninvasive cell source possessing a broad feasibility in cell therapies and tissue regeneration especially for urinary tissue engineering and also serving as original cells for disease modeling through reprogramming. However, since the biological characteristics of UDSCs have not been fully investigated yet, further basic research and practical animal studies are needed before they could be applied to the clinical therapeutics.

There are still a few issues yet to be settled. The first issue lies on the cell diversity which was displayed as line-to-line variations on both UDSCs and reprogrammed iPSCs resulting mainly from genetic background. When the experimental cells were compared with the control cells derived from another individual with different genetic background, these diversities could probably complicate the data interpretation and bring other problems such as experimental reproducibility. This issue could probably be settled through setting the experimental disease-causing mutation group and the control group originated from the same cell resource, which means that the experimental group is created by specific gene mutation on the control group through targeted genome editing technology. In such circumstance, both groups have the same genetic background except for the targeted mutation which could help to elucidate the disease-causing mechanism of the mutation. The second issue lies on the establishment of a clinical-grade cell resource of both UDSCs and reprogrammed iPSCs. This issue could probably be settled through recent technological innovations such as using integration-free reprogramming technology and xeno-free culture conditions. Great efforts have been made in making a clinical-grade cell under the guidelines of Good Manufacturing Practice and would probably benefit the patients in the near future. The third issue lies on the epigenetic memories of reprogrammed iPSCs. Which type of the original cell is chosen to reprogram usually depends on the cell accessibility, reprogramming efficiency, and the expected progression pattern of the specific disease. Several researchers have demonstrated that iPSCs reprogrammed from different original somatic cells including UDSCs exhibited distinct transcriptional and epigenetic patterns, as well as various *in vitro* differentiation potentials [58, 59]. Research also indicated that the epigenetic memory of the original cells resulted from incomplete reprogramming and the biased *in vitro* differentiation could influence the applications in disease modeling and treatment [60]. This issue could probably be settled through improving reprogramming strategies and achieving a complete pluripotency. In addition, further research is needed to elucidate the mechanisms that regulate pluripotency and to improve directed differentiation efficiency to produce the mature target cell type. Upon all these abovementioned issues, polygenic disease-specific iPSCs to recapitulate more complex diseases are facing even greater challenges.

## 5. Conclusions

In conclusion, UDSCs are a novel noninvasively obtained cell source with high proliferation ability and multiple differentiation potential and were being reprogrammed to model diseases. However, the broad and powerful application of UDSCs is yet to achieve through further investigation, both on regenerative medicine and on disease modeling after being reprogrammed.

## Figures and Tables

**Table 1:** Disease-specific iPSCs derived from urine cells.

Disease	Genetic etiology/mutation sites	Reprogramming factors	Reprogramming strategy	Major findings	Refs
Type 2 long QT syndrome	KCNH2/A561P.c7:150 648 800G>C	OSKMLN + SV40LT	Episomal vectors	A561P-UhiPSC lines were established. A561P-UhiPSCs could be differentiated into functional cardiomyocyte cells. A561P KCNH2 mutation caused a trafficking defect of the HERG channel. HERG A561P mutation increased the susceptibility to arrhythmia.	[16]
Multiple endocrine neoplasia type 1 syndrome	Men1/exon 9	OSK + miR-302-367	Episomal plasmids	MEN1-iPSC lines of male and female were established. No functional experiments were achieved.	[41, 42]
Novel heterozygous PAI-1 mutation	PAI-1/exon 4	OSKM	Sendai virus	PAI-1-iPSC line and the control line were established. No functional experiments were achieved.	[43, 44]
Hemophilia A	FVIII/intron 22 inversion	OSK + SV40LT	Episomal vectors	HA-iPSC lines were established. HA-iPSCs could be differentiated into hepatocyte-like cells in vitro. HA-iPSC-derived hepatocyte-like cells displayed FVIII deficiency.	[45]
Down syndrome	Trisomy 21	OSKM	Episomal vectors	T21-iPSC lines were established. T21-iPSCs were more sensitive to proteotoxic stress than euploid iPSCs. T21-iPSCs could be differentiated into glutamatergic neurons and cardiomyocytes. Neurons fromT21-iPSCs could fire AP similar to euploid iPSCs.	[46]
Spinal cord injury	No verification/no verification	OSKM	Sendai virus	SCI-iPSC lines were established. SCI-iPSCs could be differentiated into A2B5^+^ NPCs. A2B5^+^ NPCs could give rise to neurons and astrocytes after implantation.	[47]
Attention-deficit hyperactivity disorder	No verification/no verification	OSKM	Sendai virus	ADHD-iPSC lines were established. No functional experiments were achieved.	[48]
Dilated cardiomyopathy	No verification/no verification	OSKM	Sendai virus	DCM-iPSC lines were established. No functional experiments were achieved.	[49]
Muscular dystrophy	Dystrophin/exon deletion	OSKM	Sendai virus	MD-iPSC lines were established. MD-iPSCs lack the expression of dystrophin.	[50]
Fibrodysplasia ossificans progressiva	ALK2/R206H	OSKM/OSK + miR-302-367	Sendai virus/episomal vectors	FOP-iPSC lines were established. Differentiation efficiency into bone-forming progenitors was decreased. Expression of VEGF receptor 2 in differentiated endothelial cells was reduced. Mineralization of pericytes from FOP-hiPSCs was increased.	[51, 52]
Systemic lupus erythematosus	No verification/no verification	OSKM	Lentivirus	SLE-iPSC lines were established. No functional experiments were achieved.	[53]
Cryptorchid	INSL3/c.A178>G, ZNF214/c.A197>G, c.T383>A and c.T754>C, ZNF215/c.T108>A, c.A400>C, c.A780>T, and c.C788>T	OSKM	Lentivirus	Cryp-iPSC lines were established. Cryp-iPSCs could be differentiated into VASA^+^ germ cell.	[54]
Hypercholesterolemia	PCSK9/S127R and R104C/V114A	OSKMLN + SV40LT	Episomal vectors	PCSK9-iPSC lines were established. PCSK9-iPSCs could be differentiated into hepatocyte-like cells. PCSK9 secretion and LDL uptake were altered.	[55]
Paroxysmal kinesigenic dyskinesia	PRRT2/c.649dupC	OSKM	Retroviruses	PKD-iPSC lines were established. PKD-iPSCs could be differentiated into functional glutamatergic, dopaminergic, and motor neurons. The expression of PRRT2 was decreased in PKD-iPSCs.	[56]

The abbreviations represent a combination of reprogramming factors: O: OCT3/4; S: SOX2; K: KLF4; M: c-MYC; L: LIN28; N: NANOG.
